# Proton beam radiation therapy vs. photon radiation therapy and the overall survival of adult and pediatric patients diagnosed with sarcoma

**DOI:** 10.3389/fonc.2025.1644829

**Published:** 2025-09-18

**Authors:** Saber A. Amin, Zubair A. Mateen, Palwasha Amin, Chi Lin

**Affiliations:** ^1^ Department of Radiation Oncology, University of Nebraska Medical Center, Omaha, NE, United States; ^2^ Department of Surgery, Mayo Hospital, Lahore, Pakistan; ^3^ Department of Heart and Cardiovascular Diseases, Nebraska Medicine, Omaha, NE, United States

**Keywords:** proton beam radiation therapy, sarcoma, chordoma, rhabdomyosarcoma, chondrosarcoma, Ewing sarcoma, osteosarcoma, national cancer database

## Abstract

**Background:**

The use of proton beam radiation therapy (PBT) has increased in patients diagnosed with sarcoma. However, there is a lack of information about its survival benefit in these patients.

**Objective:**

We want to investigate the association of PBT with the overall survival (OS) of sarcoma patients.

**Methods:**

We used the National Cancer Database and assessed the OS using multivariable Cox regression analysis adjusted for age, sex, race, education, income, insurance status, histology, comorbidity score, radiation therapy dose, chemotherapy, surgery, and year of diagnosis.

**Results:**

Among the 117,694 patients, 3,573 (3%) received PBT. Patients receiving PBT had longer OS than those receiving photon radiation therapy (PRT) (HR: 0.73, CI: 0.68 -0.79). PBT was also associated with improved OS compared to PRT in chordoma (HR: 0.57, CI: 0.44-0.74), rhabdomyosarcoma (HR: 0.58, 95% CI: 0.47- 0.72), or chondrosarcoma (HR: 0.35, CI: 0.24-0.51) patients. Among patients who received surgery, PBT was associated with improved OS compared to PRT in chordoma (HR: 0.37, CI: 0.22-0.60), chondrosarcoma (HR: 0.52, CI: 0.28-0.97), or osteosarcoma (HR: 0.32, CI: 0.12-0.89) patients. Among patients with no surgery, PBT was associated with improved OS compared to PRT in chordoma (HR: 0.57, CI: 0.44-0.74), rhabdomyosarcoma (HR:0.56, CI: 0.44-0.70), chondrosarcoma (HR:0.24, CI: 0.15-0.37), osteosarcoma (HR: 0.51, CI: 0.30- 0.87), or other histology type (HR:0.76, CI: 0.66-0.86) patients.

**Conclusion:**

The use of PBT was associated with improved OS compared to photon RT in sarcoma patients. PBT was associated with improved OS in patients diagnosed with chordoma, rhabdomyosarcoma, or chondrosarcoma.

## Background

Sarcoma is a rare tumor, which represents only 0.7% of all cancer cases in the United States (1). Surgery is the mainstay of treatment (2, 3) for adult-type sarcomas, and chemotherapy is the mainstay of treatment, with surgery or radiation therapy (RT) serving for local control, for pediatric-type sarcomas such as rhabdomyosarcoma and Ewing sarcoma. The ability of sarcoma to arise from any anatomic site and the proximity to critical organs at risk make the local treatment options challenging (2, 3). In some patients, it is impossible to have a complete resection without major impairments. Surgery could be complemented or replaced by high-precision radiation therapy in these patients (3). Proton beam therapy (PBT) might be a viable and effective treatment option for some patients due to its superior dosimetry and sparring normal tissues, especially if the tumor is near the radiosensitive critical organs (4, 5).

Some evidence exists to support the clinical use of PBT in cancer patients. However, clinical evidence is still scarce. The early clinical use of PBT was mostly in pediatric cancers, including sarcomas of the skull base and spine (6–8). The strongest evidence has been in pediatric CNS tumors (9, 10). Most findings are from non-randomized, early-stage, and usually small studies with a retrospective character (11–15). However, the upcomg long-term results of prospective studies will help in understanding the role of PBT in various cancer patients (16–21).

With the increase in PBT facilities from only two in 2004 to 42 in 2022 in the U.S., the number of cancer patients who received PBT also increased from 1,206 in 20004 to 6,291 in 2022 (22). In sarcoma patients who receive surgery, RT could be used to improve local control (23). Studies also suggest that a high dose of RT could improve local control if there is a positive surgical margin, tumor recurrence, or tumor in the trunk, head, or neck (23–25). Radiation therapy is a reliable treatment option in young sarcoma patients among whom reducing toxicities such as the risk of second malignancy, fertility problems, and cognitive issues is of a great interest (26). Given that there is a lack of information about the use of PBT in sarcoma patients and its impact on overall survival (OS), we aim to investigate the association of PBT with the OS and identify factors associated with the use of PBT.

## Materials and method

### Data source

**Figure 1:**
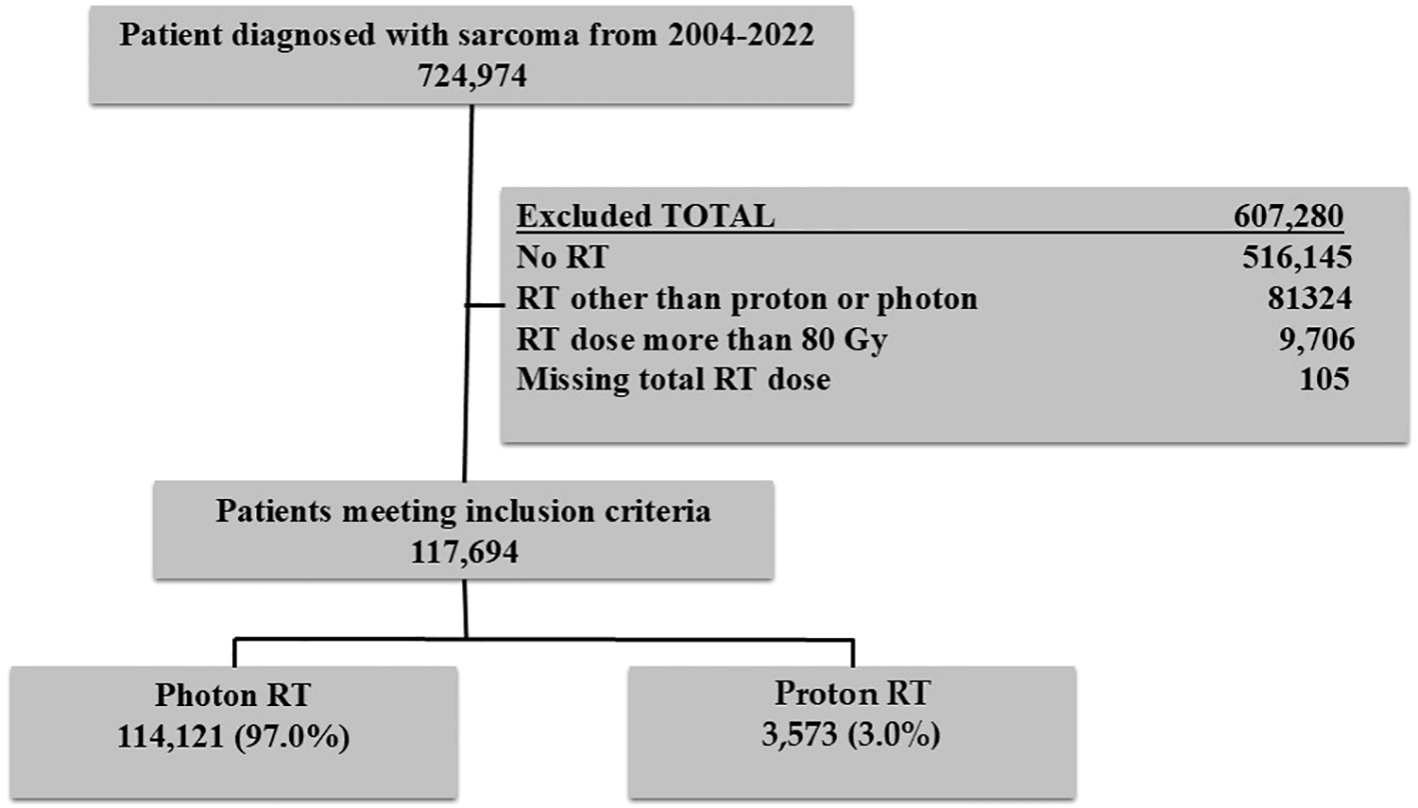
Flow chart of the study participants.

Data were extracted from the National Cancer Database (NCDB). The NCDB is the largest hospital-based cancer registry in the U.S., representing more than 70% of the cancer cases diagnosed annually. The NCDB is a consortium of more than 1500 accredited cancer hospitals that the American College of Surgeons administers. The analysis included patients diagnosed with sarcoma between 2004 and 2022. Other variables included age at diagnosis (years), insurance types (private, Medicare, Medicaid, other governmental, and no insurance), race, sex, Charlson-Deyo Comorbidity Index (0, 1, ≥2), year of diagnosis (2004–2013 or 2014–2022), neighborhood education level, median household income, and histology type. International Classification of Disease for Oncology, Third Edition (ICD-O-3) was used to determine sarcoma patients. Major histology types included Chordoma (9370,9371,9372), Rhabdomyosarcoma (8900,8901,8902,8910,8912,8920,8921), Ewing sarcoma ((9260), Chondrosarcoma (9220,9221,9231,9240,9242,9243), and other types that included the remaining histology codes. Patients who received RT dose >80Gy or who were missing RT dose were excluded. The study was exempt from review by the institutional review board as it analyzes de-identified data. Informed consent was also not needed. The flowchart of the study participants is provided in **Figure 1**.

### Statistical analysis

We reported the frequency and proportion of all the variables by PBT and photon RT. Time trends in the use of PBT were also reported by year of diagnosis. The association of the factors associated with the use of PBT was investigated by performing multivariable logistic regression analysis. The odds ratio was reported as the measure of association with the probability of receiving PBT compared to photon RT. The Kaplan–Meier survival curves were used to estimate the median survival time and compute the log-rank test to compare survival time across the PBT and photon RT groups. We used Cox proportional hazard regression models to estimate the variable’s HR and 95% CI with a focus on PBT vs. photon RT. SAS, version 9.4 (SAS Institute Inc), was used to perform analyses. All tests were 2-sided with a significance level being set at P = 0.5.

## Results

### Patient characteristics

**Figure 2:**
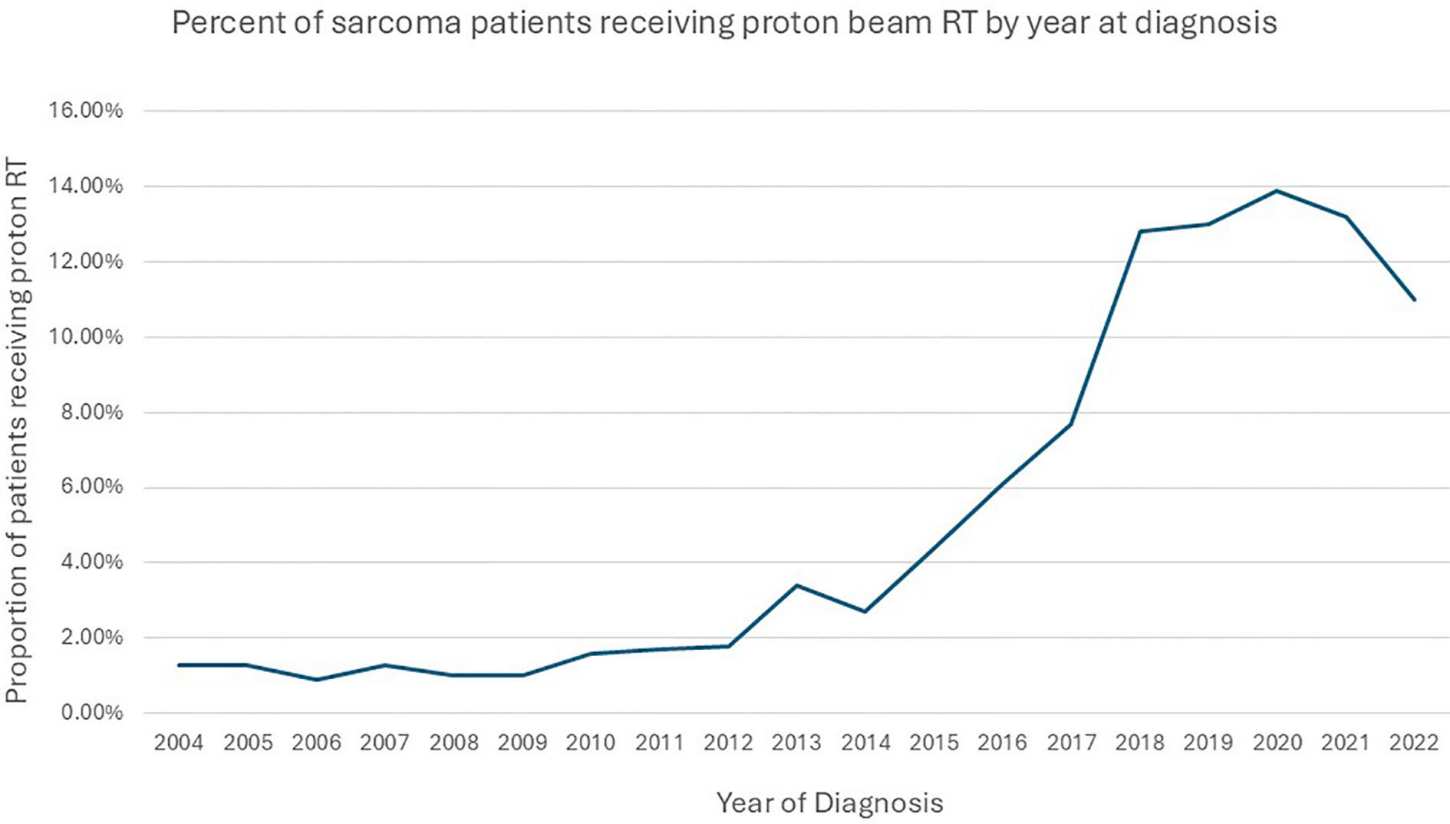
Time trends in the use of PBT by year in sarcoma patients.

Of the 117,694 study participants, 3,573 (3%) patients received PBT. Among those who received PBT, 391(10.9%) received <45 Gy,1512 (42.3%) 45–60 Gy, and 1670 (46.8%) 60–80 Gy RT dose. The use of PBT increased from 1.3% in 2004 to 11% in 2022. The use of PBT was <1% in patients younger than 18 years and 1.7% in adult patients in 2004 (**Figure 2**). In the multivariable logistic regression analysis, patients aged <18 years old, belonging to non-white non-black racial groups, and those who received 60–80 Gy were more likely to receive PBT compared to their counterparts. Patients with Medicaid, Medicare, other governmental insurance, or with no health insurance compared to (private insurance), patients with rhabdomyosarcoma, ewing sarcoma, chondrosarcoma, osteosarcoma, and other histology types compared to (chordoma) were less likely to receive PBT. In addition, patients living an area with income ≤$50353, having a comorbidity score of 1 or ≥2 compared to (zero score), a diagnosis between 2004-2013, and not receiving chemotherapy were all less likely to receive PBT. The details of the factors associated with the use of PBT are provided in **Table 1**.

**Table 1:** Baseline characteristics and factors associated with the use of PBT among patients diagnosed with sarcoma between 2004 and 2022 and were reported to the National Cancer Database (N = 117,694).

Variables		Photon RT 114,121 (97%)	Proton RT 3,573 (3%)	Total 117,694	Multivariable analysis OR (95% CI)	p
Age at diagnosis	<18	4,212(3.7%)	989(27.7%)	5,201(4.4%)	6.44 (5.58-7.43)	0.001
	≥ 18	109,909(96.3%)	2,584(72.3%)	112,493(95.6%)	Ref	
RT dose	<45Gy	35,238(30.9%)	391(10.9%)	35,629(30.3%)	0.40 (0.35-0.46)	0.001
	45-59Gy	49,429(43.3%)	1,512(42.3%)	50,941(43.3%)	Ref	
	60-80Gy	29,454(25.8%)	1,670(46.8%)	31,124(26.4%)	2.02 (1.83-2.22)	0.001
Sex	Female	57,363(50.3%)	1,668(46.7%)	59,031(50.2%)	1.01 (0.93-1.10)	0.76
	Male	56,758(49.7%)	1,905(53.3%)	58,663(49.8%)	Ref	
Race	White	95,036(84.3%)	2,841(81.7%)	97,877(84.2%)	Ref	
	African American	12,432(11%)	314(9%)	12,746(11%)	0.89 (0.77-1.02)	0.10
	Other, non-White non-Black	5,337(4.7%)	323(9.3%)	5,660(4.8%)	1.27 (1.09-1.47)	0.002
	Unknown/missing	1,316	95	1,411		
Insurance	Private	44,916(39.4%)	2,015(56.4%)	46,931(39.9%)	Ref	
	Medicaid	9,139(8%)	522(14.6%)	9,661(8.2%)	0.61 (0.48-0.77	0.001
	Medicare	52,858(46.3%)	829(23.2%)	53,687(45.6%)	0.82 (0.72-0.93)	0.003
	Other/Gov.	2,595(2.3%)	100(2.8%)	2,695(2.3%)	0.55 (0.50-0.61)	0.001
	Uninsured	4,613(4%)	107(3%)	4,720(4%)	0.86 (0.66-1.11)	0.24
Histology	Chordoma	1,125(1%)	760(21.3%)	1,885(1.6%)	Ref	
	Rhabdomyosarcoma	3,518(3%)	605(16.9%)	4,123(3.5%)	0.07 (0.06-0.10)	0.001
	Ewing sarcoma	2,064(1.8%)	224(6.3%)	2,288(1.9%)	0.04 (0.03-0.05)	0.001
	Chondrosarcoma	1,651(1.5%)	270(7.6%)	1,921(1.7%)	0.20 (0.17-0.25)	0.001
	Osteosarcoma	884(0.8%)	59(1.6%)	943(0.8%)	0.06 (0.05.10)	0.001
	All other types	104,879(91.9%)	1,655(46.3%)	106,534(90.5%)	0.02 (0.02-0.03)	0.001
Income	≤ $50,353	38,317(39%)	1,006(32%)	39,323(38.8%)	0.81 (0.73-0.89)	0.001
	>$50,353	59,883(61%)	2,141(68%)	62,024(61.2%)	Ref	
	Unknown/missing	15,921	426	16,347		
Education	≥ 10.8% NHSD	44,268(45%)	1,281(40.6%)	45,549(44.8%)	0.97 (0.88-1.07)	0.49
	<10.8% NHSD	54,147(55%)	1,870(59.4%)	56,017(55.2%)	Ref	
	Unknown/missing	15,706	422	16,128		
Charlson Comorbidity score	0	86,754(76%)	3,063(85.7%)	89,817(76.3%)	Ref	
	1	17,359(15.2%)	352(9.9%)	17,711(15%)	0.76 (0.66-0.87)	0.001
	=>2	10,008(8.8%)	158(4.4%)	10,166(8.7%)	0.69 (0.57-0.84)	0.001
Year of diagnosis	2004-2013	47,347(41.5%)	546(15.3%)	47,893(40.7%)	0.20 (0.18-0.22)	0.001
	2014-2022	66,774(58.5%)	3,027(84.7%)	69,801(59.3%)	Ref	
Chemotherapy	No	81,172(71.8%)	1,987(55.7%)	83,159(71.3%)	0.71 (0.64-0.79)	0.001
	Yes	31,875(28.2%)	1,577(44.3%)	33,452(28.7%)	Ref	
	Unknown/missing	1,074	9	1,083		
Surgery	No	72,336(64.9%)	2,182(62.5%)	74,518(64.9%)	1.01 (0.93-1.10)	0.85
	Yes	39,046(35.1%)	1,312(37.5%)	40,358(35.1%)	Ref	
	Unknown/missing	2,739	79	2,818		

NHSD, No high school degree.

### Survival outcomes

Patients who received PBT had better median OS compared to those who received photon RT. The three and five- year survival rates were 79% (95% CI: 77.4%-80.6%) and 70% (95% CI: 68%-72%) for patients who received PBT, while 61% (95% CI: 60.7%-61.3%) and 52% (95% CI: 51.7%-52.3%) for patients who received photon RT.

Patients who received PBT had better OS than those who received photon RT in the univariable Cox regression analysis (HR: 0.51, 95% CI: 0.48-0.55; p<0.001) (**Table 2**). Other factors associated with improved OS included age<18 years old, female sex, other racial groups, receiving RT dose of 60–80 Gy compared to 45–59 Gy, and not receiving chemotherapy. Black race compared to White, insurance type of Medicaid, Medicare, other governmental, and no insurance compared to private insurance, rhabdomyosarcoma, Ewing sarcoma, chondrosarcoma, osteosarcoma, and other histology types compared with chordoma, low neighborhood income level, low neighborhood education level, comorbidity score of 1 or ≥2 compared to zero comorbidity score, and no surgery compared to definitive surgery were associated with worse OS (**Table 2**). Since all variables except the year of diagnosis were significant in the univariable analyses, all except the year of diagnosis were included in the multivariable analysis. The multivariable analysis was adjusted for age at diagnosis, race, sex, insurance type, histology type, neighborhood income level, neighborhood education level, comorbidity score, use of chemotherapy, and receipt of surgery.

**Table 2:** Univariate and multivariable Cox regression analysis of the factors associated with the OS of patients diagnosed with sarcoma between 2004 and 2022.

Variables		Univariable analysis HR (95%CI)	p	Multivariable analysis HR (95%CI)	p
PRT	Photon RT	Ref		Ref	
	Proton RT	0.51(0.48-0.55)	0.001	0.73 (0.68-0.79)	0.001
Age at diagnosis	<18	0.55 (0.52-0.57)	0.001	0.50 (0.47-0.53)	0.001
	>=18	Ref		Ref	
RT dose	<45Gy	1.34 (1.31-1.36)	0.001	1.42 (1.39-1.46)	0.001
	45-59Gy	Ref		Ref	
	60-80Gy	0.94(0.92-0.96)	0.001	0.95 (0.93-0.97)	0.001
Sex	Female	0.90(0.89-0.92)	0.001	0.88 (0.86-0.90)	0.001
	Male	Ref		Ref	
Race	White	Ref		Ref	
	African American	1.12(1.09-1.15)	0.001	1.08 (1.05-1.12)	0.001
	Other, non-White non-Black	0.73(0.70-0.76)	0.001	0.82 (0.78-0.86)	0.001
Insurance	Private	Ref		Ref	
	Medicaid	1.49 (1.44-1.54)	0.001	1.39 (1.33-1.44)	0.001
	Medicare	2.30 (2.26-2.34)	0.001	2.23 (2.19-2.28)	0.001
	Other/Gov.	1.63 (1.53-1.72)	0.001	1.51(1.42-1.61)	0.001
	Uninsured	1.56 (1.49-1.63)	0.001	1.48 (1.41-1.55)	0.001
Histology	Chordoma	Ref		Ref	
	Rhabdomyosarcoma	1.70 (1.54-1.87)	0.001	1.61(1.44-1.80)	0.001
	Ewing sarcoma	1.44 (1.29-1.59)	0.001	1.33 (1.18-1.50)	0.001
	Chondrosarcoma	1.46 (1.30-1.63)	0.001	1.48 (1.31-1.67)	0.001
	Osteosarcoma	3.38 (3.01-3.80)	0.001	2.59 (2.27-2.95)	0.001
	All other types	1.99 (1.83-2.17)	0.001	1.48 (1.34-1.63)	0.001
Income	≤ $50,353	1.20 (1.18-1.22)	0.001	1.06 (1.04-1.09)	0.001
	>$50,353	Ref		Ref	
Education	≥ 10.8% NHSD	1.15 (1.12-1.17)	0.001	1.05 (1.03-1.08)	0.001
	<10.8% NHSD	Ref		Ref	
Charlson Comorbidity score	0	Ref		Ref	
	1	1.56 (1.52-1.59)	0.001	1.40 (1.37-1.44)	0.001
	≥ 2	2.12 (2.06-2.18)	0.001	1.71(1.66-1.77)	0.001
Year of diagnosis	2004-2013	1.01 (0.99-1.03)	0.26	Not included in MVA	
	2014-2022	Ref			
Chemotherapy	No	0.72 (0.71-0.74)	0.001	0.52 (0.51-0.53)	0.001
	Yes	Ref		Ref	
Surgery	No	1.44 (1.42-1.47)	0.001	1.40 (1.37-1.43)	0.001
	Yes	Ref		Ref	

NHSD, No high school degree.

Patients who received PBT had longer OS than patients who received photon RT In the multivariable analysis (HR: 0.73, 95% CI: 0.68 -0.79; p<0.001) (**Table 2**). Age <18 years old compared to ≥18 years old, female sex, and other race groups compared to White, RT dose of 60–80 Gy compared to 45–59 Gy, and no chemotherapy compared to receiving chemotherapy were other variables that were associated with improved OS in the multivariable analysis. Black race compared to White, Medicaid, Medicare, other governmental insurance, having no health insurance compared to private insurance, rhabdomyosarcoma, Ewing sarcoma, chondrosarcoma, osteosarcoma, and other histology types compared chordoma, low neighborhood income level, low neighborhood education level, comorbidity score of 1 or ≥2, compared to comorbidity score of zero, diagnosis year between 2003–2013 compared to 2014-2021, and no surgery compared to definitive surgical resection of the tumor were all associated with worse OS (**Table 2**). To reduce the imbalance noticed in **Table 1** between PBT and photon, we performed 1:1 propensity score matched analysis in which, PBT was still associated with improved OS than photon RT (HR: 0.70, 95% CI: 0.64 -0.77; p<0.001) (**Supplementary Table 1**).

In the subset analysis stratified by histology, PBT was associated with improved OS compared to photon RT in patients diagnosed with chordoma (HR: 0.57, 95% CI: 0.44-0.74; p<0.001), rhabdomyosarcoma (HR: 0.58, 95% CI: 0.47- 0.72; p<0.001), or chondrosarcoma (HR: 0.35, 95% CI: 0.24-0.51; p<0.001) (**Table 3**).

**Table 3:** Multivariable Cox regression analysis of PBT vs. photon RT stratified by histology.

Histology	HR (95%) PBT vs photon RT	P
Chordoma	0.57 (0.44-0.74)	0.001
Rhabdomyosarcoma	0.58 (0.47- 0.72)	0.001
Ewing sarcoma	0.86 (0.64-1.15)	0.31
Chondrosarcoma	0.35 (0.24-0.51)	0.001
Osteosarcoma	0.69 (0.43-1.12)	0.13
Other	0.91 (0.83- 1.00)	0.06

In the analysis stratified by surgery, PBT was associated with improved OS compared to photon RT in patients diagnosed with chordoma (HR: 0.37, 95% CI: 0.22-0.60; p<0.001), chondrosarcoma (HR: 0.52, 95% CI: 0.28-0.97; p<0.04), or osteosarcoma (HR: 0.32, 95% CI: 0.12-0.89; p<0.001) in patients who received definitive surgery of the tumor (**Table 4**). Among patients who did not receive surgery, PBT was associated with improved OS in patients diagnosed with chordoma (HR: 0.57, 95% CI: 0.44-0.74; p<0.001), rhabdomyosarcoma (HR:0.56, 95% CI: 0.44-0.70; p<0.001), chondrosarcoma (HR:0.24, 95% CI: 0.15-0.37; p<0.001), osteosarcoma (HR: 0.51, 95% CI: 0.30- 0.87; p<0.001), or other histology type (HR:0.76, 95% CI: 0.66-0.86; p<0.001) (**Table 4**).

**Table 4:** Multivariable Cox regression analysis of PBT vs. photon RT stratified by surgery and histology.

Surgery		
Histology type	HR (95% CI)	P
Chordoma	0.37 (0.22-0.60)	0.001
Rhabdomyosarcoma	0.60 (0.32-1.13)	0.11
Ewing sarcoma	0.89 (0.42-1.86)	0.75
Chondrosarcoma	0.52 (0.28-0.97)	0.04
Osteosarcoma	0.32 (0.12-0.89)	0.03
Other	1.06 (0.92-1.22)	0.44

No surgery		
Chordoma	0.57 (0.44-0.74)	0.001
Rhabdomyosarcoma	0.56 (0.44-0.70)	0.001
Ewing sarcoma	0.85 (0.62- 1.16)	0.31
Chondrosarcoma	0.24 (0.15-0.37)	0.001
Osteosarcoma	0.51 (0.30- 0.87)	0.001
Other	0.76 (0.66-0.86)	0.001

When the analysis was restricted to RT dose 60–80 Gy and stratified by surgery, PBT was associated with improved OS compared to photon RT only in patients diagnosed with chordoma (HR: 0.36, 95% CI: 0.20-0.68; p<0.002) among those who received definitive surgery (**Table 5**). Among those who did not receive surgery, PBT was associated with improved OS compared to photon RT in patients diagnosed with chordoma (HR: 0.56 (0.41- 0.78) or chondrosarcoma (HR: 0.26, 95% CI: 0.15-0.44; p<0.001) (**Table 5**). The sample size for other dose categories and histology types was small and no analysis was performed.

**Table 5:** Multivariable Cox regression analysis stratified by surgery and histology for RT dose 60-80.

Surgery and RT dose 60–80 Gy		
Histology type	HR (95% CI)	P
Chordoma	0.36 (0.20-0.68)	0.002
Chondrosarcoma	0.67 (0.34- 1.32)	0.25

No Surgery and RT dose 60–80 Gy		
Chordoma	0.56 (0.41- 0.78)	0.001
Chondrosarcoma	0.26 (0.15-0.44)	0.001

When the analysis was stratified by RT dose and histology, we had enough sample size only for rhabdomayosarcoma and other histology types among patients who received RT dose <45 Gy. Proton RT was associated with improved OS than photon RT in patients diagnosed with rhabdomayosarcoma (HR:0.45, 95% CI: 0.27-0.72; P<0.001) but not in other histology type (HR:0.91. 95% CI: 0.72-1.16; p=0.45). Among patients who received RT dose 45–59 Gy, PBT was associated with improved OS in rhabdomayosarcoma patients (HR: 0.62, 95% CI: 0.48-0.79; p<0.001) and other histology type (HR: 0.75, 95% CI: 0.64-0.87; p<0.001) but not in Ewing sarcoma patients (HR: 0.95, 95% CI: 0.68-1.32; p=0.75). There was not enough sample size for the remaining histology types. Among patients who received RT dose 60–80 Gy, PBT was associated with improved OS in chordoma (HR:0.53, 95% CI: 0.40-0.70; p<0.001) and chondrosarcoma (HR: 0.34, 95% CI: 0.22-0.52; p<0.001) but not in all other types (HR: 1.11, 95% CI: 0.96-1.28; p=0.15) (**Supplementary Table 2**).

In a subset analysis of the subtypes of rhabdomayosarcoma (NOS, embryonal, and alveolar), PBT was associated with improved OS compared to photon RT in NOS (HR: 0.41, 95% CI: 0.23-0.73; p<0.001), embryonal (HR: 0.48, 95% CI: 0.33-0.71; p<0.001), and alveolar (HR: 0.75, 95% CI: 0.55-1.00; p=0.05).

While a higher proportion of patients received chemotherapy in the proton RT group than photon RT 1,577 (44.3%) vs. 31875 (28.2%), the overall results of proton RT vs. photon RT did not change when we stratified by chemotherapy. Proton RT was associated with improved OS than photon RT in patients who received chemotherapy (HR:0.73, 95% CI: 0.66-0.81; p<0.001) and those who did not receive chemotherapy (HR:0.72, 95% CI: 0.64-0.80; p<0.001).

We wanted to perform an analysis stratify by histology and neoadjuvant and adjuvant RT. However, there was not enough sample size for the neoadjuvant group. Among the adjuvant RT, the OS results did not change from the general Cox regression model.

## Discussion

The current study is the largest to evaluate PBT’s association with sarcoma patients’ OS in real-world settings. It is the first study to report that sarcoma patients who received PBT had longer OS than those who received photon RT. In the current study, more than 50% of the patients who received PBT were younger than 40 years old. This is important since PBT has been reported to improve normal tissue sparing and to reduce cognitive issues (26).

In the analysis stratified by histology, the use of PBT was associated with improved OS compared to photon RT only in patients diagnosed with chordoma, rhabdomyosarcoma, or chondrosarcoma. Among patients who received surgery, PBT was associated with improved OS in patients diagnosed with chordoma, chondrosarcoma, or osteosarcoma. Among patients who did not receive surgery, the use of PBT was associated with improved OS in patients diagnosed with chordoma, rhabdomyosarcoma, chondrosarcoma, osteosarcoma, and other histology types. When the analysis was restricted to an RT dose of 60–80 Gy, among patients who received surgery, PBT was associated with improved OS compared to photon RT only in patients diagnosed with chordoma. Among those who did not receive surgery, PBT was associated with improved OS compared to photon RT in patients diagnosed with chordoma or chondrosarcoma.

The survival findings reported in our study are similar to some other studies of PBT in sarcoma patients. The five-year survival rate of 85% in chordoma patients in the current study, is comparable to the five-year survival rate reported in some published case series (27–30). The five-year survival rates in these series were,86%, 74.6%, 66.7%, and 80.5% (27–30). The five-year and two-year survival rates of 85% and 95% of chondrosarcoma patients in our study are similar to the five-year survival rate of 83.5%, and a two-year survival rate of 93.5% reported by previous studies (27, 31). The four-year survival rate of 89% for chondrosarcoma patients in our study is better than the four-year survival rate of 72% reported in a case series of patients treated with PBT who had either chordoma or chondrosarcoma (32).

The three and five-year survival rates of 78% and 71% for rhabdomyosarcoma patients in our study are comparable to 81% and 77% reported in clinical trials (33, 34). The five-year survival rate of 71% in our study is slightly higher than the 64% (35) reported by a previous study. These studies were case series and did not have a comparison group and included only pediatric patients (34, 35). The one, two, three, four, and five-year survival rates of 95%, 85%, 78%, 73%, and 71% of rhabdomyosarcoma patients in our study are also comparable with 93%, 85%, 80%, 71%, and 82% reported by a systematic review and meta-analysis that investigated the efficacy and safety of PBT in rhabdomyosarcoma patients (36).

The improved OS associated with the use of PBT compared to photon RT in chordoma and chondrosarcoma patients might be due to a higher dose of RT being delivered safely when PBT was used in these patients. Most chordoma and chondrosarcoma patients who received 60–80 Gy were treated with PBT. In chordoma specifically, 90% of PBT-treated patients received 60–80 Gy versus 44.5% in the photon RT cohort. Thus, the apparent overall survival advantage with PBT could reflect dose escalation, as these tumors are relatively radioresistant. Nevertheless, in our study PBT remained associated with improved OS even when analyses were restricted to patients receiving 60–80 Gy. The survival benefit of PBT in chordoma and chondrosarcoma patients was also not driven by the benefit of surgery as PBT was associated with better OS compared to photon RT when the analysis was stratified by surgery.

Our study found that, among patients who did not undergo surgery, proton beam therapy (PBT) was associated with improved overall survival across chordoma, chondrosarcoma, rhabdomyosarcoma, osteosarcoma, and pooled “other” sarcoma histologies. Ewing sarcoma was the only histology in which PBT was not associated with improved OS, irrespective of surgical status. These results suggest that PBT may confer a survival advantage in the presence of gross disease, including tumors traditionally considered radioresistant. The signal in the non-surgical subgroup is notable given longstanding skepticism about radiotherapy for radioresistant histologies such as osteosarcoma. Although osteosarcoma is generally less responsive to radiation and surgery is preferred when feasible, radiotherapy can improve local control—and potentially survival—for unresectable axial disease or after resections with positive margins (4). Consistent with this, the ongoing Children’s Oncology Group trial (AOST2032; NCT05691478) includes radiotherapy for unresectable disease, postoperative positive margins, and selected metastatic lesions.

An argument could be made that the improved OS associated with the use of PBT may be due to the imbalance between PBT and photon RT reported in **Table 1**. However, PBT was associated with improved OS compared to photon in the propensity matched analysis. PBT was also associated with improved OS after stratifying by important factors such as surgery, histology, and age at diagnosis. These results provide indication that the survival benefit associated with the use of PBT is not due to the difference in other factors between the two groups of RT.

The use of PBT increased from 1.4% in 2004 to 12% in 2022. The increase in the use of PBT is due to many reasons, the most important being the increase in the number of PBT facilities in operation (37–39). In 2004, only two facilities were operating, while in 2022, 42 facilities offered treatment with PBT. The proportion of sarcoma patients receiving PBT increased by more than 800% from 45 in 2004 to 394 in 2022, coinciding with an almost 20 times increase in facilities from 2004 to 2022. More than 85% of the patients who received PBT were diagnosed between 2014 and 2022. The acceptance of PBT use by oncologists and the growing number of prospective clinical trials are some additional contributing factors (38, 39).

Pediatric patients were more likely to receive PBT compared to adult sarcoma patients. The higher likelihood could be due to the stronger clinical evidence and a stronger belief in the benefit of the use of PBT in pediatric cancers, including CNS tumors and sarcomas of the skull base and spine (6, 40, 41).

The higher odds of using PBT in patients with private insurance compared to Medicare, Medicaid, and no insurance is an indication of the challenge and barriers in accessing advanced treatment techniques in patients without private insurance. Patients with Medicare had the lowest odds of receiving PBT, which may be due to the strict requirements for the approval of PBT. An alternative payment model has been introduced for Medicare recipients, which focuses on reducing Medicare expenditure and overuse of treatment with unproven benefits while preserving the quality of care (42). It has to be noted that access to proton therapy is extremely difficult worldwide, with significant healthcare costs for society.

Patients living in an area with ≤$50,353 neighborhood income level were also less likely to receive PBT. Previous studies of non-small cell lung cancer and other cancers have reported similar findings (43–45). The finding, together with racial disparity, is an indication of the presence of structural racism and disparity in access to modern treatment across the U.S. It is also an elaboration that access to innovative cancer treatments is not uniform. Much work is needed to overcome socioeconomic barriers in access to modern treatments such as PBT. These issues must be addressed at the healthcare policy level. Patients with a comorbidity score of 1 or ≥2 were less likely to receive PBT compared to a score of zero, a probable indication that PBT has been used in patients who are healthier, live longer, and are more likely to benefit from it.

Despite being the first and largest study of PBT use in sarcoma patients, it has several limitations. Important limitations include the retrospective nature of NCDB, which makes the database prone to errors. Some additional limitations include the lack of information about acute and late toxicity, recurrence, reirradiation, performance status, cause of death, and disease progression. Only 3% of the patients received PBT, which makes this population unique with different characteristics, such as the ability to have access to new and modern treatments at a high cost. Due the different characteristics of the PBT group, the role of different time periods, different age groups, and different dose levels can not be ingored when assessing the impact of PBT on the OS of these patients.

Nevertheless, this comprehensive study of the NCDB is the first study to report the survival benefit associated with the receipt of PBT in children and adult sarcoma patients using a large database. Despite a sharp increase in the number of PBT facilities, the use of PBT in sarcoma patients remains low compared to the number of patients who receive photon RT. In conclusion, the use of PBT was associated with improved OS compared to photon RT. In the stratified analysis by histology, the use of PBT was associated with improved OS in patients diagnosed with chordoma, rhabdomyosarcoma, or chondrosarcoma. In the subset analysis among patients who received surgery on the tumor, the use of PBT was associated with improved OS in patients diagnosed with Chordoma, chondrosarcoma, or osteosarcoma. In the no-surgery group, only in Ewing sarcoma patients, PBT was not associated with improved OS. When the analysis was stratified by surgery and restricted only to 60–80 Gy, PBT use was associated with improved OS only in chordoma patients who received surgery, while its use was associated with improved OS in chordoma and chondrosarcoma patients who did not receive surgery. Finally, our study revealed that the use of PBT among sarcoma patients has increased, but there is still a large gap between the number of patients who should receive PBT and patients who are receiving PBT. The increase in the number of PBT facilities is a step in the right direction, but much more is needed to adopt a broader use of PBT.

## Data Availability

The data analyzed in this study is subject to the following licenses/restrictions: The data will be provided upon a reasonable request from the corresponding author. The NCBD is not a publicly available database. Requests to access these datasets should be directed to clin@unmc.edu.
