# Supplementary material for: Proton beam radiation therapy vs. photon radiation therapy and the overall survival of adult and pediatric patients diagnosed with sarcoma
Source: Front Oncol. 2025 Sep 18;15:1644829. doi: 10.3389/fonc.2025.1644829 (PMC12488416; doi:10.3389/fonc.2025.1644829)
Supplement: Supplementary file 1 [file Table1.docx]

Supplemental Table 1. Overall survival of sarcoma patients using 1:1 (PBT=2,955, photon RT=2,955) propensity score matched analysis

| Variables | | Multivariable analysis  HR (95%CI) | p |
| --- | --- | --- | --- |
| PRT | Photon RT | Ref |  |
|  | Proton RT | 0.70 (0.64-0.77) | 0.001 |
| Age at diagnosis | <18 | 0.50 (0.43-0.58) | 0.001 |
|  | >=18 | Ref |  |
| RT dose | <45Gy | 1.56 (1.35-1.79) | 0.001 |
|  | 45-59Gy | Ref |  |
|  | 60-80Gy | 1.03 (0.92-1.15) | 0.60 |
| Sex | Female | 0.91 (0.83-0.99) | 0.03 |
|  | Male | Ref |  |
| Race | White | Ref |  |
|  | African American | 0.99 (0.85-1.17) | 0.95 |
|  | Other, non-White non-Black | 0.79 (0.66-0.94) | 0.01 |
| Insurance | Private | Ref |  |
|  | Medicaid | 1.23 (1.07-1.42) | 0.01 |
|  | Medicare | 1.92 (1.72-2.14) | 0.001 |
|  | Other/Gov. | 1.57(1.55-2.13) | 0.004 |
|  | Uninsured | 1.12 (0.87-1.45) | 0.38 |
| Histology | Chordoma | Ref |  |
|  | Rhabdomyosarcoma | 1.64(1.31-2.05) | 0.001 |
|  | Ewing sarcoma | 1.39 (1.09-1.78) | 0.01 |
|  | Chondrosarcoma | 1.14 (0.93-1.41) | 0.21 |
|  | Osteosarcoma | 1.87 (1.34-2.60) | 0.001 |
|  | All other types | 1.81 (1.57-2.10) | 0.001 |
| Income | <=$50353 | 1.03 (0.92-1.15) | 0.59 |
|  | >$50,353 | Ref |  |
| Education | >=10.8% NHSD | 1.01 (0.90-1.12) | 0.94 |
|  | <10.8% NHSD | Ref |  |
| Charlson Comorbidity score | 0 | Ref |  |
|  | 1 | 1.29 (1.12-1.48) | 0.001 |
|  | =>2 | 1.46 (1.20-1.78) | 0.001 |
| Chemotherapy | No | 0.57 (0.51-0.64) | 0.001 |
|  | Yes | Ref |  |
| Surgery | No | 1.51 (1.37-1.66) | 0.001 |
|  | Yes | Ref |  |

NHSD: No high school degree
