# Supplementary material for: Proton beam radiation therapy vs. photon radiation therapy and the overall survival of adult and pediatric patients diagnosed with sarcoma
Source: Front Oncol. 2025 Sep 18;15:1644829. doi: 10.3389/fonc.2025.1644829 (PMC12488416; doi:10.3389/fonc.2025.1644829)
Supplement: Supplementary file 2 [file Table2.docx]

Supplemental Table 2. Multivariable Cox regression analysis of PBT vs. photon RT stratified by RT dose

| Histology types | HR (95% CI) | P |
| --- | --- | --- |
| RT dose <45 Gy | | |
| Rhabdomyosarcoma | 0.45 (0.27-0.72) | 0.001 |
| All other types | 0.91 (0.72-1.16) | 0.45 |
| RT dose 45-59 Gy | | |
| Rhabdomyosarcoma | 0.62 (0.48-0.79) | 0.001 |
| Ewing sarcoma | 0.95 (0.68-1.32) | 0.75 |
| All other types | 0.75 (0.64-0.87) | 0.001 |
| RT dose 60-80 Gy | | |
| Chordoma | 0.53(0.40-0.70) | 0.001 |
| Chondrosarcoma | 0.34 (0.22-0.52) | 0.001 |
| All other types | 1.11 (0.96-1.28) | 0.15 |
